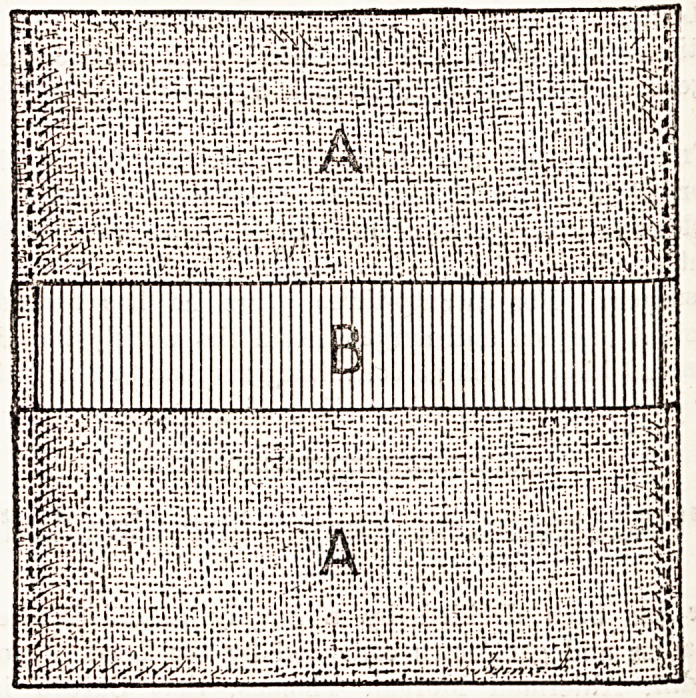# Institutional Needs

**Published:** 1913-05-10

**Authors:** 


### Institutional Needs.

### THE COMPLETE PAD OR DRAW-SHEET SUBSTITUTE.

An extremely ingenious nursing appliance has been designed by a lady of St. Leonards, which takes the form of a substitute for a draw-sheet. The "Complete Pad" may be made by taking a piece of common butter muslin of suitable dimensions and folding the ends over in such a way that they almost meet in the middle. The edges on either side A are then stitched, a bag thus being formed which has an opening or slot across the middle B, as illustrated in the accompanying drawing. Into this opening is introduced a piece of good mackintosh sheeting, the size of the bag, followed by a covering of cheap absorbent wool. The advantage of this device is that as soon as the Pad is soiled, the mackintosh can be removed through the opening, and the rest of the appliance destroyed. The Pad, which was exhibited by Dr. Vickerman Newland at a recent meeting of the Hastings Division of the B.M.A., was 18 inches square, and it was stated that the cost, exclusive of the mackintosh, was about 2d. The size used must necessarily be determined by the requirements of the case.

### "BYNO" LECITHIN.

(Manufactured by Messrs. Allen and Hanburys, Ltd.)

This preparation is a new departure in the treatment of nervous exhaustion. It is a combination of lecithin—a phosphorised body which enters largely into the composition of the nervous system—with cinchona and nux vomica alkaloids in a solution of "Bynin" or liquid malt. Recent clinical experience and physiological investigation show that the preparation seems to be especially indicated in those cases which cannot assimilate a phosphorus compound not so closely allied chemically to the natural combination in the body as lecithin.

## Figures and Tables

**Figure f1:**